# Crystal structure and Hirshfeld surface analysis of (*Z*)-4-(4-hy­droxy­benzyl­idene)-3-methyl­isoxazol-5(4*H*)-one

**DOI:** 10.1107/S2056989018007867

**Published:** 2018-06-08

**Authors:** Wissem Zemamouche, Rima Laroun, Noudjoud Hamdouni, Ouarda Brihi, Ali Boudjada, Abdelmadjid Debache

**Affiliations:** a Laboratoire de Cristallographie, Département de Physique, Université Mentouri-Constantine, 25000 Constantine, Algeria; b Laboratoire de Synthèse de Molécules, d’Intérêts Biologiques, Département de Chimie, Université Mentouri-Constantine, 25000 Constantine, Algeria

**Keywords:** crystal structure, isoxazole, hy­droxy­benzyl­idene, *Z* configuration, hydrogen bonding, offset π–π inter­actions, Hirshfeld surface

## Abstract

The title compound, C_11_H_9_NO_3_, contains an isoxazole and a hy­droxy­benzyl­idene ring, which are inclined to each another by 3.18 (8)°. There is an intra­molecular C—H⋯O contact forming an *S*(7) ring. In the crystal, mol­ecules stack head-to-tail in columns along the *b*-axis direction, linked by offset π–π inter­actions [inter­centroid distances of 3.676 (1) and 3.723 (1) Å]. The columns are linked by O—H⋯O and O—H⋯N hydrogen bonds, forming layers parallel to the *ab* plane. The layers are linked by C—H⋯O hydrogen bonds, forming a supra­molecular three-dimensional framework. An analysis of the Hirshfeld surfaces points to the importance of the O—H⋯O and O—H⋯N hydrogen bonding in the packing mechanism of the crystal structure.

## Abstract 2

In the crystal of the title compound, mol­ecules stack head-to-tail in columns along the *b*-axis direction, and are linked by offset π–π inter­actions [inter­centroid distances of 3.676 (1) and 3.723 (1) Å].

## Chemical context   

The isoxazole ring system is a component of many natural and medicinally active mol­ecules that exhibit inter­esting biological activities (Wang *et al.*, 2012 ▸). Isoxazole derivatives have been shown to possess anti­convulsant (Balalaie *et al.*, 2000 ▸), anti­fungal (Santos *et al.*, 2010 ▸), HDAC inhibitory (Conti *et al.*, 2010 ▸), analgesic (Kano *et al.*, 1967 ▸), anti­microbial (Padmaja *et al.*, 2009 ▸), anti­tuberculosis (Lee *et al.*, 2009 ▸), anti­mycobacterial (Mao *et al.*, 2010 ▸) and many other biological properties. They are also used for the treatment of leishmaniasis (Changtam *et al.*, 2010 ▸) and for the treatment of patients with active arthritis (Suryawanshi *et al.*, 2012 ▸). Furthermore, the isoxazole unit can be used as the basis for the design and construction of merocyanine dyes, which are used in optical recording and non-linear optical research (Zhang *et al.*, 2011 ▸). In the present study, we report on the synthesis, crystal structure and Hirshfeld surface analysis of the title isoxazole derivative.
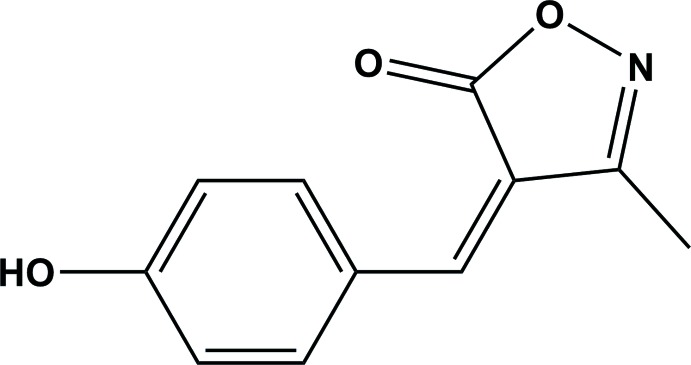


## Structural commentary   

**Figure 1:**
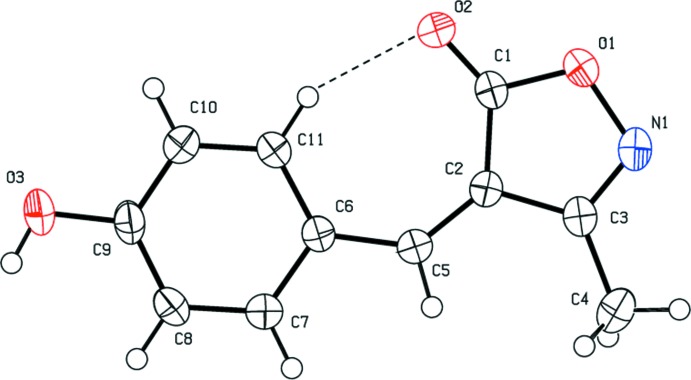
The mol­ecular structure of the title compound, with atom labelling and displacement ellipsoids drawn at the 50% probability level. The intra­molecular C—H⋯O contact (see Table 1 ▸) is shown as a dashed line.

The mol­ecular structure of the title compound is shown in Fig. 1 ▸. The mol­ecule is composed of an isoxazole ring (O1/N1/C1–C3) that is almost coplanar with the benzene ring (C6–C11) of the 4-hy­droxy­benzyl­idene substituent; the two rings are inclined to each other by 3.18 (8)°. The configuration about the C2=C5 bond is *Z*, and within the mol­ecule there is a short intra­molecular C11—H11⋯O2 contact (Table 1 ▸), forming an S(7) ring motif. The bond lengths and bond angles agree well with those observed for a similar compound, the 2-hy­droxy­benzyl­idene analogue, (*Z*)-4-(2-hy­droxy­benzyl­idene)-3-methyl­isoxazol-5(4*H*)-one (Cheng *et al.*, 2009 ▸). Here the hydroxyl group is in the *ortho* position, compared to the *para* position in the title compound.

## Supra­molecular features   

**Figure 2:**
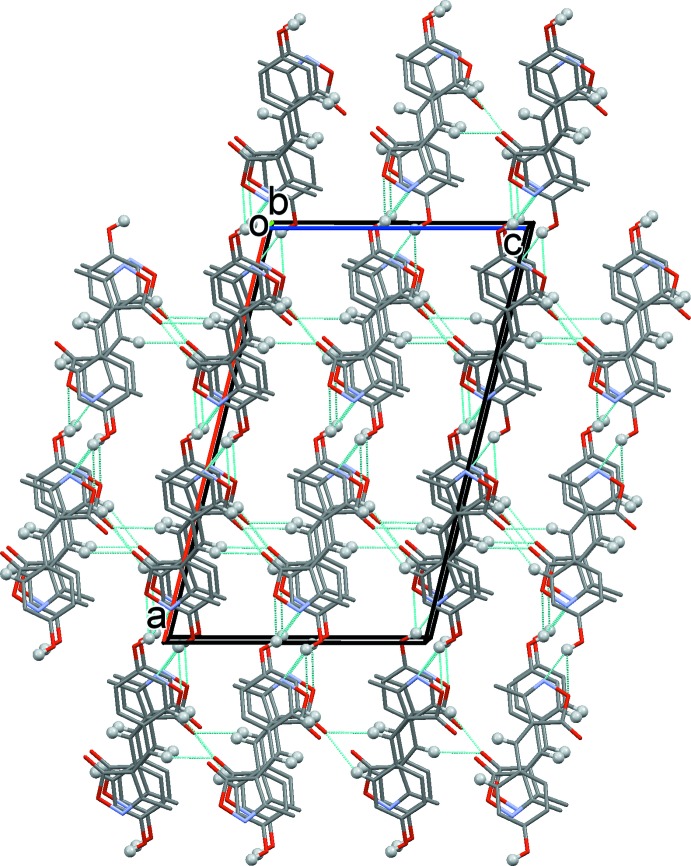
A view along the *b* axis of the crystal packing of the title compound. Only the H atoms (grey balls) involved in hydrogen bonding (see Table 1 ▸) have been included.

In the crystal, mol­ecules stack head-to-tail along the *b*-axis direction (Fig. 2 ▸), and are linked by offset π–π inter­actions: *Cg*1⋯*Cg*2^iii,iv^ inter­centroid distances are 3.676 (1) and 3.723 (1) Å, inter­planar distances are 3.426 (1) and 3.489 (1) Å, slippages are 1.287 and 1.458 Å with the rings inclined to each other by 3.18 (8)°; symmetry codes: (iii) −*x* + 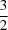, −*y* + 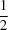, −*z* + 1; (iv) −*x* + 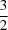, −*y* − 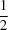, −*z* + 1. The mol­ecular columns are linked by O—H⋯O and O—H⋯N hydrogen bonds (Table 1 ▸), forming layers parallel to (001). The layers are linked by C—H⋯O hydrogen bonds, forming a supra­molecular three-dimensional framework (Table 1 ▸ and Fig. 2 ▸).

## Analysis of the Hirshfeld surfaces   

Additional insight into the inter­molecular inter­actions was obtained from an analysis of the Hirshfeld surface (Spackman & Jayatilaka, 2009 ▸) and the two-dimensional fingerprint plots (McKinnon *et al.*, 2007 ▸). The program *CrystalExplorer* (Turner *et al.*, 2017 ▸) was used to generate Hirshfeld surfaces mapped over *d*_norm_, *d*_e_ and the electrostatic potential for the title compound.

**Figure 3:**
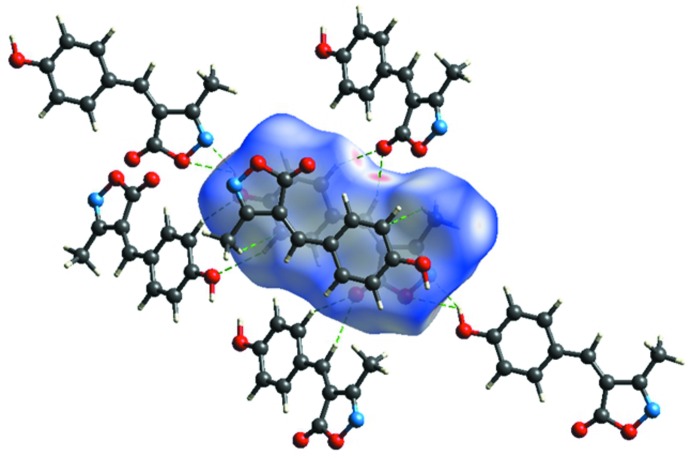
A view of the Hirshfeld surface mapped over *d*_norm_, with neighbouring inter­actions shown as green dashed lines.

**Figure 4:**
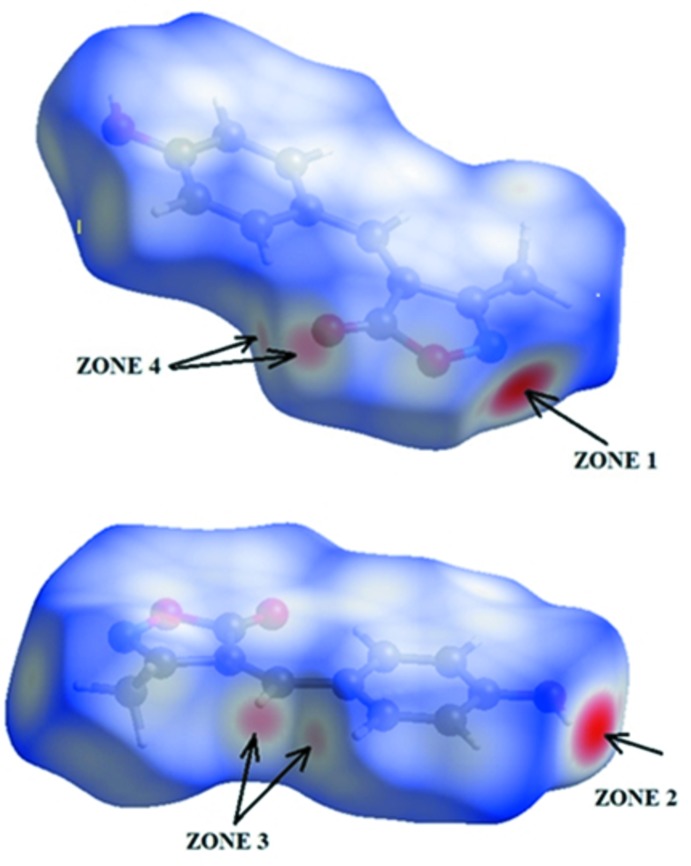
Two views of the Hirshfeld surface mapped over *d*_norm_.

**Figure 5:**
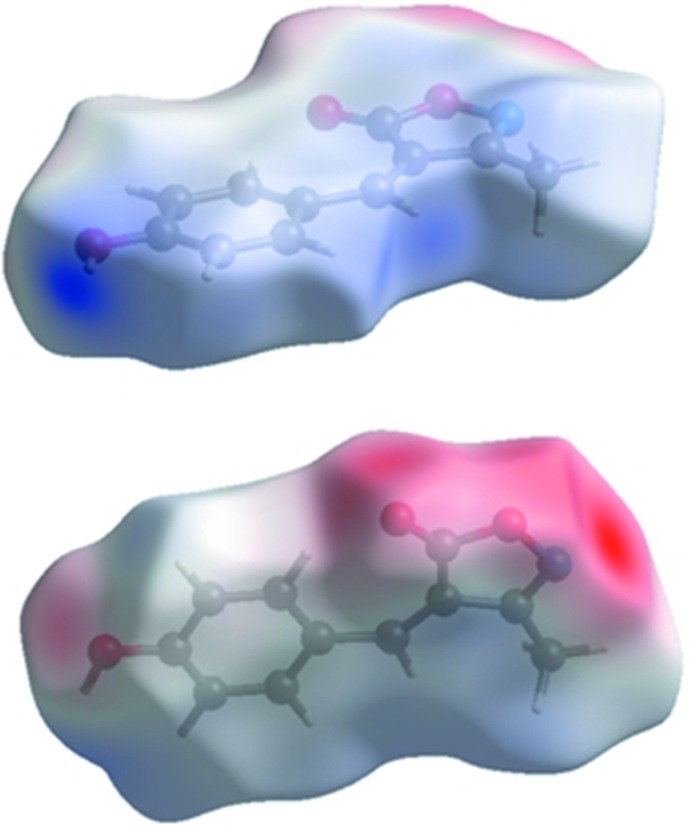
Two views of the Hirshfeld surface mapped over the electrostatic potential.

The analysis of the Hirshfeld surface mapped over *d*_norm_ is shown in Fig. 3 ▸. The O3—H3⋯O1^i^ and O3—H3⋯N1^i^ inter­actions between the corresponding donor and acceptor atoms are visualized as bright-red spots on both sides (zones 1 and 2) of the Hirshfeld surfaces (Fig. 4 ▸). Two other red spots exist, corresponding to the C5—H5⋯O2^ii^ and C7—H7⋯O^ii^ inter­actions (Fig. 4 ▸, zones 3 and 4); these are considered to be weak inter­actions by comparing them to the sum of the van der Waals radii. The donors and acceptors of inter­molecular hydrogen bonds appear as blue and red regions, respectively, around the participating atoms on the Hirshfeld surface mapped over the calculated electrostatic potential (Fig. 5 ▸).

**Figure 6:**
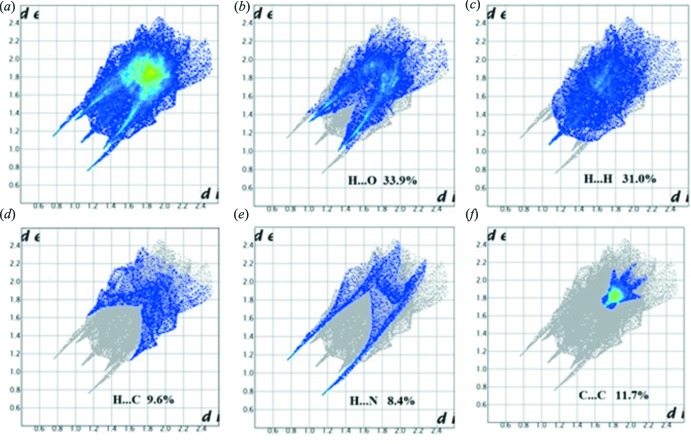
Two-dimensional fingerprint plots: (*a*) overall, and delineated into contributions from different contacts: (*b*) H⋯O/O⋯H, (*c*) H⋯H, (*d*) H⋯C/C⋯H, (*e*) H⋯NN⋯H, (*f*) C⋯C.

The overall two-dimensional fingerprint plot is illustrated in Fig. 6 ▸ *a*, and the H⋯O/O⋯H, H⋯H, C⋯H/H⋯C, and N⋯H/H⋯N contacts are illustrated in Fig. 6 ▸ *b*–*f*, respectively. The H⋯O/O⋯H contacts (Fig. 6 ▸ *b*) account for 33.9% of the Hirshfeld surface, representing the largest contribution and is displayed on the fingerprint plots by a pair of short spikes at *d*_e_ + *d*_i_ = 2.3 Å. This distance is *ca* 0.5 Å shorter than the sum of the van der Waals radii of the individual atoms, which means it is a very strong inter­action. A contribution of 31.0% was found for the inter­atomic H⋯H contacts (Fig. 6 ▸ *c*), with a distinctive peak in the fingerprint plot at *d*_e_ + *d*_i_ = 2.2 Å; the van der Waals radius for this inter­action is 2.4 Å. The H⋯C/C⋯H contacts (9.6% contribution; Fig. 6 ▸ *d*) are indicated by a pair of short peaks at *d*_e_ + *d*_i_ = 2.7 Å, equal to the sum of the van der Waals radii. The H⋯N/N⋯H contacts (Fig. 6 ▸ *e*), which account for only 8.4% of the Hirshfeld surface, are displayed on the fingerprint plot as a pair of long spikes at *d*_e_ + *d*_i_ = 2.0 Å. This distance differs by *ca* 0.7 Å from the sum of the van der Waals radii, which means it is the strongest inter­action present. The C⋯C contacts (Fig. 6 ▸ *f*), which account for 11.7% of the Hirshfeld surface with *d*_e_ + *d*_i_ = 3.4 Å, indicate the presence of π–π stacking.

## Database survey   

A search of the Cambridge Structural Database (CSD, V3.59, last update February 2018; Groom *et al.*, 2016 ▸) for 4-substituted 3-methyl-isoxzol-5(4*H*)-ones gave 22 hits. Of these, six compounds involve a benzyl­idene substituent. The configuration about the C=C bond is *Z* in all six compounds and the benzene ring is inclined to the isoxazole ring by angles as small as 1.14° in (*Z*)-4-benzyl­idene-3-methyl­isoxazol-5(4*H*)-one (MBYIOZ01; Chandra *et al.*, 2012 ▸) compared to *ca* 11.59° in (*Z*)-4-(4-meth­oxy­benzyl­idene)-3-methyl-1,2-oxazol-5(4*H*)-one (YIMWIC; Saikh *et al.*, 2013 ▸). The most relevant structure is the 2-hy­droxy­benzyl­idene analogue, *viz*. (*Z*)-4-(2-hy­droxy­benzyl­idene)-3-methyl­isoxazol-5(4*H*)-one (AJESAK; Cheng *et al.*, 2009 ▸), in which the two rings are inclined to each other by *ca* 6.53°, compared to 3.18 (8)° in the title compound. The *Z* configuration of all six mol­ecules indicates that there is an intra­molecular C—H⋯O contact present forming an *S*(7) ring motif, as in the title compound (Fig. 1 ▸ and Table 1 ▸).

## Synthesis and crystallization   

4-Hy­droxy­benzaldehyde (1 mmol), hydroxyl­amine hydro­chloride (1 mmol), ethyl­aceto­acetate (1 mmol) and K_2_CO_3_ (5 ml) were mixed in a 25 ml flask equipped with a magnetic stirrer. The mixture was refluxed in 5 ml of water for 1 h (the reaction was monitored by TLC). On completion of the reaction, the mixture was gradually poured into ice-cold water. Stirring was maintained for a few minutes and the obtained solid was filtered and purified by crystallization from ethanol (yield 83%), yielding pale-yellow needle-like crystals on slow evaporation of the solvent.

## Refinement   

Crystal data, data collection and structure refinement details are summarized in Table 2 ▸. The hydroxyl H atom was located in a difference-Fourier map and freely refined. The C-bound H atoms were included in calculated positions and treated as riding: C—H = 0.93–0.96 Å with *U*_iso_(H) = 1.5*U*_eq_(C-meth­yl) and 1.2*U*_eq_(C) for other H atoms.

## Supplementary Material

Crystal structure: contains datablock(s) I, global. DOI: 10.1107/S2056989018007867/zp2027sup1.cif

Structure factors: contains datablock(s) I. DOI: 10.1107/S2056989018007867/zp2027Isup2.hkl

Supporting information file. DOI: 10.1107/S2056989018007867/zp2027Isup3.cml

CCDC reference: 1845567

Additional supporting information:  crystallographic information; 3D view; checkCIF report

## Figures and Tables

**Table 1:** Hydrogen-bond geometry (Å, °)

*D*—H⋯*A*	*D*—H	H⋯*A*	*D*⋯*A*	*D*—H⋯*A*
C11—H11⋯O2	0.93	2.15	2.989 (2)	149
O3—H3*O*⋯O1^i^	0.86 (2)	2.41 (2)	2.9119 (18)	118 (2)
O3—H3*O*⋯N1^i^	0.86 (2)	2.00 (2)	2.7984 (19)	154 (2)
C5—H5⋯O2^ii^	0.93	2.47	3.3655 (17)	162
C7—H7⋯O2^ii^	0.93	2.55	3.4038 (19)	152

Symmetry codes: (i) 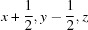; (ii) 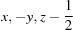.

**Table 2:** Experimental details

Crystal data	
Chemical formula	C_11_H_9_NO_3_
*M*_r_	203.19
Crystal system, space group	Monoclinic, *C*2/*c*
Temperature (K)	293
*a*, *b*, *c* (Å)	21.191 (2), 7.2352 (11), 12.9569 (14)
β (°)	103.920 (11)
*V* (Å^3^)	1928.2 (4)
*Z*	8
Radiation type	Mo *K*α
μ (mm^−1^)	0.10
Crystal size (mm)	0.36 × 0.23 × 0.11

Data collection	
Diffractometer	Agilent Technologies Xcalibur, Eos
Absorption correction	Multi-scan (*CrysAlis PRO*; Agilent, 2013 ▸)
*T*_min_, *T*_max_	0.551, 1.000
No. of measured, independent and observed [*I* > 2σ(*I*)] reflections	4536, 1891, 1465
*R*_int_	0.021
(sin θ/λ)_max_ (Å^−1^)	0.617

Refinement	
*R*[*F* ^2^ > 2σ(*F* ^2^)], *wR*(*F* ^2^), *S*	0.040, 0.113, 1.04
No. of reflections	1891
No. of parameters	142
H-atom treatment	H atoms treated by a mixture of independent and constrained refinement
Δρ_max_, Δρ_min_ (e Å^−3^)	0.19, −0.13

Computer programs: *CrysAlis PRO* (Agilent, 2013 ▸), *SIR92* (Altomare *et al.*, 1994 ▸), *PLATON* (Spek, 2009 ▸), *Mercury* (Macrae *et al.*, 2008 ▸), *SHELXL2018/3* (Sheldrick, 2015 ▸) and *publCIF* (Westrip, 2010 ▸).

## supplementary crystallographic information

### Crystal data

**Table:**

C_11_H_9_NO_3_	*F*(000) = 848
*M**_r_* = 203.19	*D*_x_ = 1.400 Mg m^−^^3^
Monoclinic, *C*2/*c*	Mo *K*α radiation, λ = 0.71073 Å
*a* = 21.191 (2) Å	Cell parameters from 1714 reflections
*b* = 7.2352 (11) Å	θ = 4.1–32°
*c* = 12.9569 (14) Å	µ = 0.10 mm^−^^1^
β = 103.920 (11)°	*T* = 293 K
*V* = 1928.2 (4) Å^3^	Needle, pale yellow
*Z* = 8	0.36 × 0.23 × 0.11 mm

### Data collection

**Table:**

Agilent Technologies Xcalibur, Eos diffractometer	1891 independent reflections
Radiation source: Enhance (Mo) X-ray Source	1465 reflections with *I* > 2σ(*I*)
Graphite monochromator	*R*_int_ = 0.021
ω scans	θ_max_ = 26.0°, θ_min_ = 3.2°
Absorption correction: multi-scan (CrysalisPro; Agilent, 2013)	*h* = −26→23
*T*_min_ = 0.551, *T*_max_ = 1.000	*k* = −8→8
4536 measured reflections	*l* = −15→12

### Refinement

**Table:**

Refinement on *F*^2^	Secondary atom site location: difference Fourier map
Least-squares matrix: full	Hydrogen site location: mixed
*R*[*F*^2^ > 2σ(*F*^2^)] = 0.040	H atoms treated by a mixture of independent and constrained refinement
*wR*(*F*^2^) = 0.113	*w* = 1/[σ^2^(*F*_o_^2^) + (0.0595*P*)^2^] where *P* = (*F*_o_^2^ + 2*F*_c_^2^)/3
*S* = 1.04	(Δ/σ)_max_ = 0.001
1891 reflections	Δρ_max_ = 0.19 e Å^−^^3^
142 parameters	Δρ_min_ = −0.13 e Å^−^^3^
0 restraints	Extinction correction: (SHELXL-2018/3; Sheldrick, 2015), Fc^*^=kFc[1+0.001xFc^2^λ^3^/sin(2θ)]^-1/4^
Primary atom site location: structure-invariant direct methods	Extinction coefficient: 0.0020 (6)

### Special details

**Table:**

Geometry. All esds (except the esd in the dihedral angle between two l.s. planes) are estimated using the full covariance matrix. The cell esds are taken into account individually in the estimation of esds in distances, angles and torsion angles; correlations between esds in cell parameters are only used when they are defined by crystal symmetry. An approximate (isotropic) treatment of cell esds is used for estimating esds involving l.s. planes.

### Fractional atomic coordinates and isotropic or equivalent isotropic displacement parameters (Å^2^)

**Table:**

	*x*	*y*	*z*	*U*_iso_*/*U*_eq_	
O1	0.62276 (5)	0.14571 (16)	0.60644 (8)	0.0492 (4)	
O2	0.72284 (6)	0.0722 (2)	0.69282 (8)	0.0626 (4)	
O3	0.99352 (6)	−0.2162 (2)	0.60528 (10)	0.0613 (4)	
H3O	1.0132 (11)	−0.237 (3)	0.5558 (16)	0.092 (8)*	
N1	0.58554 (6)	0.16143 (18)	0.49783 (10)	0.0448 (4)	
C1	0.68443 (8)	0.0886 (2)	0.60805 (12)	0.0392 (4)	
C2	0.68735 (7)	0.06174 (19)	0.49745 (11)	0.0308 (3)	
C3	0.62309 (7)	0.11381 (19)	0.43784 (12)	0.0347 (4)	
C4	0.59837 (9)	0.1192 (2)	0.32016 (13)	0.0521 (5)	
H4A	0.558271	0.187052	0.302307	0.078*	
H4B	0.591061	−0.004561	0.293277	0.078*	
H4C	0.629819	0.178707	0.289026	0.078*	
C5	0.73584 (7)	0.00370 (18)	0.45337 (10)	0.0321 (4)	
H5	0.723867	0.000592	0.379466	0.039*	
C6	0.80178 (7)	−0.05397 (19)	0.49675 (11)	0.0309 (3)	
C7	0.83942 (7)	−0.0968 (2)	0.42463 (11)	0.0374 (4)	
H7	0.820742	−0.088293	0.352088	0.045*	
C8	0.90318 (8)	−0.1508 (2)	0.45834 (12)	0.0407 (4)	
H8	0.927260	−0.178127	0.409006	0.049*	
C9	0.93153 (7)	−0.1646 (2)	0.56651 (13)	0.0391 (4)	
C10	0.89502 (8)	−0.1264 (2)	0.63930 (12)	0.0414 (4)	
H10	0.913860	−0.136911	0.711693	0.050*	
C11	0.83143 (7)	−0.0732 (2)	0.60553 (11)	0.0374 (4)	
H11	0.807415	−0.049326	0.655426	0.045*	

### Atomic displacement parameters (Å^2^)

**Table:**

	*U*^11^	*U*^22^	*U*^33^	*U*^12^	*U*^13^	*U*^23^
O1	0.0342 (7)	0.0808 (8)	0.0351 (6)	0.0182 (6)	0.0132 (5)	0.0031 (5)
O2	0.0413 (7)	0.1180 (11)	0.0271 (6)	0.0283 (7)	0.0056 (5)	−0.0015 (6)
O3	0.0259 (6)	0.1075 (11)	0.0490 (8)	0.0214 (6)	0.0061 (6)	−0.0077 (7)
N1	0.0290 (8)	0.0631 (8)	0.0410 (8)	0.0122 (7)	0.0057 (6)	0.0029 (6)
C1	0.0271 (8)	0.0580 (9)	0.0335 (8)	0.0104 (7)	0.0091 (7)	0.0017 (7)
C2	0.0256 (8)	0.0372 (7)	0.0289 (7)	0.0031 (6)	0.0050 (6)	0.0010 (6)
C3	0.0274 (8)	0.0405 (7)	0.0363 (8)	0.0049 (7)	0.0076 (7)	0.0005 (6)
C4	0.0392 (10)	0.0710 (11)	0.0397 (9)	0.0134 (9)	−0.0027 (8)	−0.0004 (8)
C5	0.0294 (8)	0.0403 (7)	0.0263 (7)	0.0033 (6)	0.0061 (6)	−0.0002 (6)
C6	0.0258 (8)	0.0373 (7)	0.0297 (7)	0.0036 (6)	0.0067 (6)	−0.0021 (6)
C7	0.0324 (9)	0.0516 (9)	0.0283 (7)	0.0051 (7)	0.0075 (6)	−0.0001 (6)
C8	0.0297 (9)	0.0582 (9)	0.0374 (8)	0.0057 (7)	0.0141 (7)	−0.0051 (7)
C9	0.0225 (8)	0.0503 (9)	0.0435 (8)	0.0067 (7)	0.0062 (7)	−0.0045 (7)
C10	0.0323 (9)	0.0588 (9)	0.0304 (8)	0.0081 (7)	0.0025 (7)	−0.0033 (7)
C11	0.0292 (8)	0.0531 (9)	0.0310 (8)	0.0083 (7)	0.0093 (6)	−0.0032 (6)

### Geometric parameters (Å, º)

**Table:**

O1—C1	1.3660 (19)	C5—C6	1.4370 (19)
O1—N1	1.4426 (15)	C5—H5	0.9300
O2—C1	1.2054 (18)	C6—C7	1.401 (2)
O3—C9	1.3417 (18)	C6—C11	1.4053 (19)
O3—H3O	0.86 (2)	C7—C8	1.373 (2)
N1—C3	1.2860 (19)	C7—H7	0.9300
C1—C2	1.462 (2)	C8—C9	1.389 (2)
C2—C5	1.357 (2)	C8—H8	0.9300
C2—C3	1.445 (2)	C9—C10	1.384 (2)
C3—C4	1.489 (2)	C10—C11	1.368 (2)
C4—H4A	0.9600	C10—H10	0.9300
C4—H4B	0.9600	C11—H11	0.9300
C4—H4C	0.9600		
			
C1—O1—N1	109.59 (11)	C6—C5—H5	113.2
C9—O3—H3O	112.2 (14)	C7—C6—C11	117.27 (13)
C3—N1—O1	107.22 (11)	C7—C6—C5	117.34 (13)
O2—C1—O1	118.53 (14)	C11—C6—C5	125.39 (13)
O2—C1—C2	134.54 (15)	C8—C7—C6	121.68 (13)
O1—C1—C2	106.93 (12)	C8—C7—H7	119.2
C5—C2—C3	124.58 (13)	C6—C7—H7	119.2
C5—C2—C1	131.96 (13)	C7—C8—C9	119.66 (14)
C3—C2—C1	103.46 (13)	C7—C8—H8	120.2
N1—C3—C2	112.79 (13)	C9—C8—H8	120.2
N1—C3—C4	119.70 (14)	O3—C9—C10	117.26 (14)
C2—C3—C4	127.50 (14)	O3—C9—C8	122.99 (14)
C3—C4—H4A	109.5	C10—C9—C8	119.75 (14)
C3—C4—H4B	109.5	C11—C10—C9	120.49 (14)
H4A—C4—H4B	109.5	C11—C10—H10	119.8
C3—C4—H4C	109.5	C9—C10—H10	119.8
H4A—C4—H4C	109.5	C10—C11—C6	121.12 (14)
H4B—C4—H4C	109.5	C10—C11—H11	119.4
C2—C5—C6	133.54 (13)	C6—C11—H11	119.4
C2—C5—H5	113.2		
			
C1—O1—N1—C3	0.71 (16)	C1—C2—C5—C6	0.4 (3)
N1—O1—C1—O2	178.29 (14)	C2—C5—C6—C7	−176.83 (15)
N1—O1—C1—C2	−1.37 (16)	C2—C5—C6—C11	4.0 (3)
O2—C1—C2—C5	1.7 (3)	C11—C6—C7—C8	−1.6 (2)
O1—C1—C2—C5	−178.74 (15)	C5—C6—C7—C8	179.15 (14)
O2—C1—C2—C3	−178.13 (19)	C6—C7—C8—C9	0.2 (2)
O1—C1—C2—C3	1.45 (16)	C7—C8—C9—O3	−179.90 (15)
O1—N1—C3—C2	0.28 (17)	C7—C8—C9—C10	1.0 (2)
O1—N1—C3—C4	−178.93 (13)	O3—C9—C10—C11	−179.90 (14)
C5—C2—C3—N1	179.09 (14)	C8—C9—C10—C11	−0.7 (2)
C1—C2—C3—N1	−1.08 (17)	C9—C10—C11—C6	−0.7 (2)
C5—C2—C3—C4	−1.8 (2)	C7—C6—C11—C10	1.8 (2)
C1—C2—C3—C4	178.06 (14)	C5—C6—C11—C10	−178.97 (14)
C3—C2—C5—C6	−179.81 (14)		

### Hydrogen-bond geometry (Å, º)

**Table:**

*D*—H···*A*	*D*—H	H···*A*	*D*···*A*	*D*—H···*A*
C11—H11···O2	0.93	2.15	2.989 (2)	149
O3—H3*O*···O1^i^	0.86 (2)	2.41 (2)	2.9119 (18)	118 (2)
O3—H3*O*···N1^i^	0.86 (2)	2.00 (2)	2.7984 (19)	154 (2)
C5—H5···O2^ii^	0.93	2.47	3.3655 (17)	162
C7—H7···O2^ii^	0.93	2.55	3.4038 (19)	152

Symmetry codes: (i) *x*+1/2, *y*−1/2, *z*; (ii) *x*, −*y*, *z*−1/2.
