# Crystal structure and Hirshfeld surface analysis of (*Z*)-4-(4-hy­droxy­benzyl­idene)-3-methyl­isoxazol-5(4*H*)-one. Corrigendum

**DOI:** 10.1107/S2056989022007265

**Published:** 2022-07-22

**Authors:** Wissame Zemamouche, Rima Laroun, Noudjoud Hamdouni, Ouarda Brihi, Ali Boudjada, Abdelmadjid Debache

**Affiliations:** a Laboratoire de Cristallographie, Département de Physique, Université Mentouri-Constantine, 25000 Constantine, Algeria; b Laboratoire de Synthèse de Molécules, d’Intérêts Biologiques, Département de Chimie, Université Mentouri-Constantine, 25000 Constantine, Algeria

## Abstract

In the paper by Zemamouche *et al.* [ *Acta Cryst.* (2018), E**74**, 926–930], there is an error in the name of the first author.

## Abstract 2

Corrigendum to *Acta Cryst.* (2018), E**74**, 926–930.

The name of the first author in the paper by Zemamouche *et al.* (2018 ▸) is incorrect and should be ‘Wissame Zemamouche’ as given above.
